# Fertility Preservation in Children and Adolescents With Cancer: Pilot of a Decision Aid for Parents of Children and Adolescents With Cancer

**DOI:** 10.2196/10463

**Published:** 2018-11-28

**Authors:** Catherine Allingham, Lynn Gillam, Maria McCarthy, Margaret Zacharin, Sadunee Jayasuriya, Yves Heloury, Lisa Orme, Michael Sullivan, Michelle Peate, Yasmin Jayasinghe

**Affiliations:** 1 Department of Obstetrics & Gynaecology The Royal Women’s Hospital University of Melbourne Parkville Australia; 2 Department of Paediatric & Adolescent Gynaecology The Royal Children’s Hospital Parkville Australia; 3 Melbourne School of Population and Global Health University of Melbourne Parkville Australia; 4 Children's Bioethics Centre The Royal Children's Hospital Parkville Australia; 5 Department of Psychology The Royal Children's Hospital Parkville Australia; 6 Murdoch Children's Research Institute Parkville Australia; 7 Department of Endocrinology The Royal Children's Hospital Parkville Australia; 8 Monash University Clayton Australia; 9 Department of Urology The Royal Children's Hospital Parkville Australia; 10 Children's Cancer Centre The Royal Children's Hospital Parkville Australia

**Keywords:** adolescent, cancer, decision aid, fertility preservation, pediatric, shared decision making, Values Clarification Exercise

## Abstract

**Background:**

Future infertility is a significant concern for survivors of childhood and adolescent cancer. Children and adolescents may have the opportunity to undergo fertility preservation (FP) procedures (which preserve gonadal tissue or gametes for future use) prior to the cancer treatment. However, the decision is very complex, as it is often made by parents as proxy decision makers at the time of cancer diagnosis, and is time-sensitive (needing to occur before the cancer treatment begins). Furthermore, FP procedures in children and adolescents are experimental and cannot guarantee future fertility. An uninformed decision may result in future decision regret.

**Objective:**

This study aimed to assess the acceptability, usability, and feasibility of a Web-based FP decision aid (DA) in parents of children and adolescents with cancer and clinicians. Fertility knowledge and decision regret were compared in families who reviewed the DA compared with those who did not.

**Methods:**

The Web-based DA was developed according to the International Patient Decision Aid Standards. A cross-sectional study of parents of patients with cancer, who discussed fertility, and clinicians at a tertiary children’s hospital was undertaken. The acceptability, usability, and feasibility of the DA were assessed using a pre-post survey design. Measures included the validated Decision Regret Scale, a purpose-designed fertility-related knowledge scale, questions regarding satisfaction with the DA, and open-ended responses for additional feedback. Furthermore, clinicians involved in FP were also invited to review the DA.

**Results:**

We enrolled 34 parents and 11 clinicians in this study. Participants who reviewed the DA (15 parents and 11 clinicians) expressed satisfaction with its content and functionality. Parents reported an improved understanding of cancer treatments, infertility, and FP procedures and did not report greater decision regret after DA review. Most parents (13/15, 86%) would recommend the DA to other parents. All clinicians had a consensus that this was a valid and relevant information source for all involved in fertility care.

**Conclusions:**

It is an international standard of care to discuss the impact of cancer treatment on fertility before cancer treatment. This is the first fertility DA for parents of children and adolescents with cancer and is found to be relevant and acceptable by parents and clinicians. This DA has the potential to help support parents to make informed fertility-related decisions for their children and adolescents. However, future research is needed to assess the impact of the DA on prospective decision making.

## Introduction

Australia has one of the highest incidences of childhood cancer worldwide, with >1500 children (0-12 years) and adolescent-young-adult patients (13-25 years) newly diagnosed annually [1,2]. Improvements in care have seen the 5-year survival rate surpass 83%, after which, the lifetime survival is comparable to that of their healthy peers [2,3]. Attention must be given to the late effects of cancer diagnosis and treatment in this growing population of survivors [2].

Common cancer treatments (alkylating chemotherapies and radiotherapy) may have gonadotoxic effects that can damage the reproductive system, resulting in infertility or sterility [4-8]. The risk to fertility is variable and difficult to exactly predict [9,10]. For some patients, this risk is negligible; for others, however, infertility may be almost certain [8,9]. Treatment regimen and dosage, sex, age at diagnosis, pubertal status, and disease are factors that may affect the risk of infertility. Survivors of childhood cancer regard infertility as one of their greatest concerns [7,8,10]. Fertility preservation (FP) procedures may be offered to patients at risk of infertility when medically appropriate [7].

Research regarding the application and efficacy of FP in humans is ongoing [8,11]. Gender and pubertal status determine the availability and accessibility of FP. For females, oocyte and embryo cryopreservation are the most effective means of preserving fertility but not possible in children [7,11]. In addition, ovarian tissue cryopreservation is available, although still considered experimental, with only 130 live births recorded to date [12]. For males, semen cryopreservation is currently the only viable FP procedure available. Preservation of immature testicular tissue is yet to be proven successful in humans [13]. Therefore, ovarian and testicular tissue harvesting procedures are usually only offered to children and adolescents under special governance [8,14].

Many young people are simply too young or feel too overburdened to make the fertility decision themselves and are glad that their parents take the initiative [15,16]. Overall, 48% of young people and 42% of parents experience posttraumatic stress symptoms around the diagnosis. Parental consent for FP decisions is usually required for all children under the age of 18 years (because of vulnerability), with only 33% of boys aged <12 years able to appropriately comprehend fertility information [17]. The importance of parental input is further highlighted that even in young adults (age≤25 years), parents contribute to fertility decisions in 82% of cases. Thus, a large weight of responsibility sits with parents. The potential procedure-related risks, time delays in cancer treatment, and the potential message of false hope regarding cancer survival or the success of FP procedures must be considered [18]. Of concern, is the potential misinterpretation of risks and unrealistic expectations of FP success by patients and their parents [11,18]. The clarification of these factors is of vital importance and may be aided through the provision of balanced and understandable information.

Much of the information regarding FP is new to parents, and the involvement of patients in the decision-making process is variable [8,19]. Patients and families have limited time to consider their options as FP procedures are best undertaken prior to the commencement of gonadotoxic cancer treatments, soon after a cancer diagnosis [8,10]. In addition, there is often no clearly preferable decision, with each FP option having its own inherent risks and benefits that need to be considered with respect to personal values [20]. Thus, the decision to forgo or to pursue FP is difficult, and in this ethically complex scenario, decision makers require decision support.

Decision aids (DA) are educational tools designed to complement clinician counseling and facilitate difficult preference-sensitive decisions [21]. DAs have been shown across a range of health care choices to reduce the decisional conflict (a measure of uncertainty), increase decision satisfaction and knowledge, and minimize future regret, without increasing harm [21]. DAs are now considered to be the “gold standard” approach to shared decision making for complex health care decisions [21,22]. Considering the complexity of FP decision making in the pediatric setting, a DA could provide standardized, evidence-based decision support for parents of pediatric patients with cancer. To the best of our knowledge currently no FP DA is available for use in this clinical setting. Thus, this study aimed to develop and assess the acceptability, usability, and feasibility of a Web-based FP DA for parents of children with cancer who had previously made a fertility discussion as part of their clinical care. In addition, this study compared fertility knowledge and decision regret around their decision in families who reviewed the DA compared with those who did not. Finally, this study aimed to assess the clinician acceptance of the DA by its perceived usefulness and whether they would recommend its use in the clinical practice.

## Methods

### Participants and Study Design

This study used a cross-sectional pre-post survey design. Parents of patients with cancer (aged 0-18 years) diagnosed between December 2010 and December 2015 at The Royal Children’s Hospital, Melbourne, were invited to participate. In addition, clinicians involved in oncofertility (gynecologists, endocrinologists, oncologists, ethicists, pediatric surgeons, and *in vitro* fertilization specialists) were invited to review the DA. All parents had previously discussed their child’s fertility with their clinical team. This retrospective study design aimed to minimize the risk to new patients, which is typical for DA pilot studies [9,23]. Ethics approval was obtained from The Royal Children’s Hospital Human Research Ethics Committee (36016A).

### Participant Population

Parents were eligible for participation if their child’s cancer diagnosis occurred within 5 years before December 2015; the child was not on active treatment; they previously had an FP discussion; were proficient in English; and had consented to be contacted for future research. Families where the child was palliative or deceased were excluded. Furthermore, families were excluded if the treating oncologist felt it was clinically inappropriate for them to be contacted for research purposes. The child’s risk of infertility was classified as low (<20%), medium (20%-80%), or high (>80%), according to previously published risk tables [9].

### Clinician Population

Of 24 invited clinicians, 46% (11/24) consented to participate in this study and completed a post-DA review survey. Of these, 82% (9/11) of the clinicians were involved in FP consultations and were from the disciplines of gynecology, endocrinology, urology, oncology, and clinical ethics.

### Procedure

All eligible parents were provided with an invitation pack by the researcher, containing an introductory letter, information sheet, consent form, and pre-DA questionnaire. Once consenting parents (1 parent per family) completed the questionnaire, they were given access to the Web-based DA and the post-DA questionnaire.

Clinicians involved in fertility consultations and oncological care were approached either in person or through email. If clinicians consented, they either met for an informal discussion and completed the survey or reviewed the DA online and completed the survey online.

### Data Analysis

Data were analyzed using SPSS V22 (IBM Corp, Armonk, NY, USA). Descriptive statistics (means, ranges, and SDs) were calculated to describe sample characteristics and response rates and to assess DA acceptability. In addition, *t* tests were used to compare normally distributed data. Furthermore, thematic analysis was conducted on open-ended responses.

### Decision Aid

Development of the electronic DA was theoretically guided by Coulter et al [24] and the International Patient Decision Aid Standards, an evidence-based theoretical model for effective behavioral interventions [23,25]. The DA content was informed by two formalized information needs assessments, which were conducted at the Royal Children’s Hospital [26,27]; and input from FP Taskforce consumer group. The DA was developed using the WordPress Content Management System (a software application that allows users to design and manage Web-content and materials). The DA has 11 chapters with 22 pages (Multimedia Appendix 1). Where appropriate, content was divided according to the patients’ gender. Furthermore, medical illustrations and infographics were included to help quantify the risks of various outcomes and enhance patients’ understanding [28].

**Figure 1:**
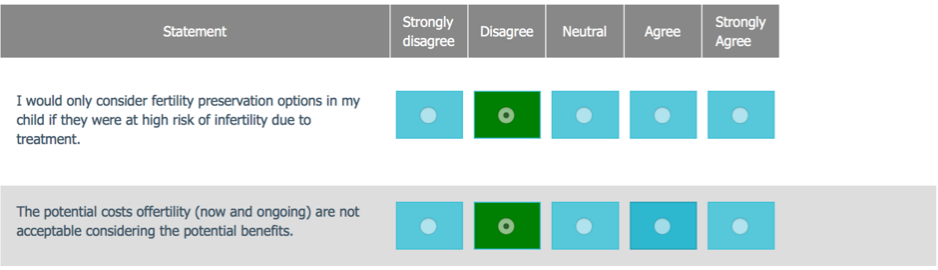
Example questions from the values clarification exercise.

**Figure 2:**
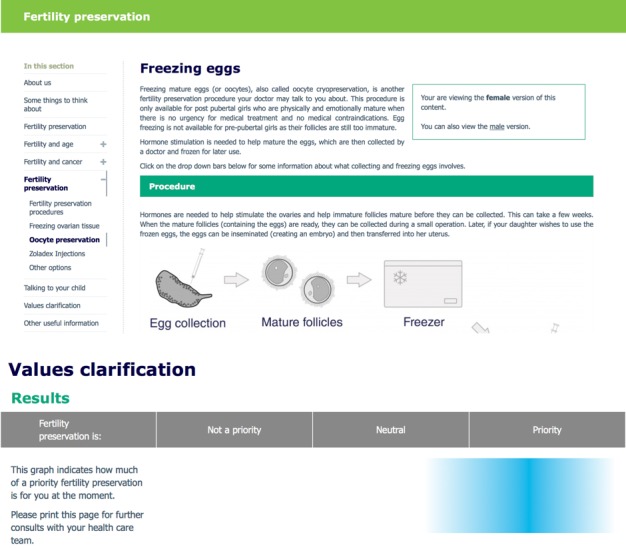
Decision aid and values clarification results bar.

A novel Web-based Values Clarification Exercise (VCE) was developed for this DA. Questions were designed to help parents clarify the importance of their child’s fertility in the context of cancer diagnosis and treatment planning. Parents rated sex- and age-specific statements on a Likert-type scale with responses ranging from “strongly disagree” to “strongly agree.” Results were scored from −2 to +2. Higher scores (eg, +2) indicated that fertility was considered a priority, whereas lower scores (eg, −2) indicated that fertility was not a priority; 0 was considered neutral. Figure 1 provides an example of the VCE questions. Parents were provided with a results summary bar (Figure 2), where their mean score represented the priority of FP for the parent and SD represented the variability around that score. The mean VCE score is plotted as a percentage of 100, where “not a priority” ranges from 0% to 33%, “neutral” ranges from 34% to 67%, and “priority” ranges from 68% to 100%. Color spread is calculated using SD, adjusted to a range between 10 and 50 points, and spread from the central score in both directions.

### Survey Measures

Questionnaires were adapted from those previously used in similar studies [25,29]. Multimedia Appendix 2 outlines the outcome measures assessed. The clinician survey included a question of whether the clinician would recommend the DA to patients and an open-ended question about future improvements and thoughts.

## Results

### Response Rates

**Figure 3:**
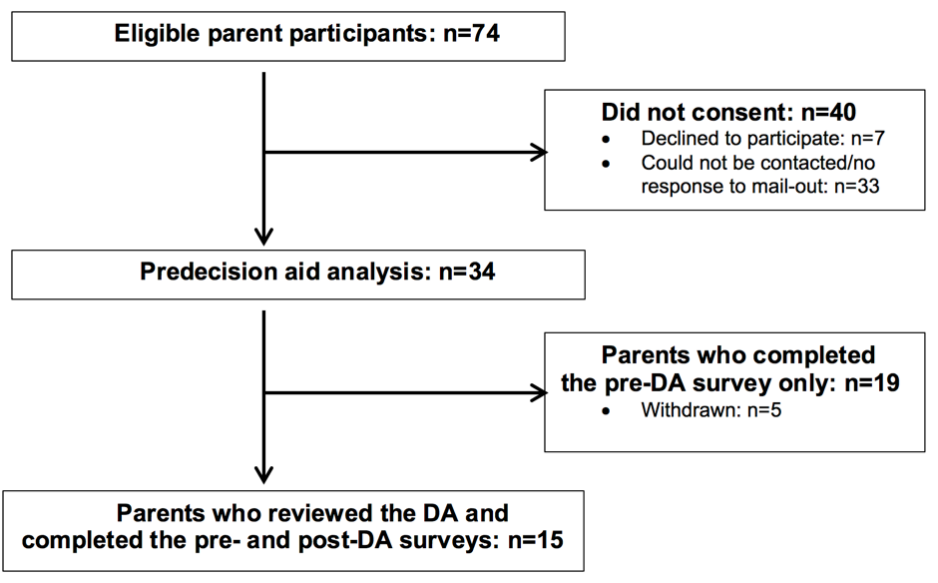
Parent participant recruitment flowchart.

In this study, 74 families were eligible for participation. Of these, 34 parents consented to participate and completed the pre-DA questionnaire (survey 1). Then, 19 parents withdrew after completing survey 1, citing time constraints (n=5), or did not respond to follow-up (n=14). Subsequently, 15 parents reviewed the DA and completed the post-DA questionnaire (Figure 3).

### Characteristics of Parents Who Reviewed the Decision Aid

The mean age of parents was 43 years. Compared with parents who only completed survey 1 (n=19), those who reviewed the DA (n=15) were more likely to be in part-time or full-time employment and have a higher level of education (Table 1). In addition, the distribution of infertility risk differed markedly between the 2 groups (Table 2). Otherwise, the 2 groups were similar with respect to demographics and concerns around their child’s future fertility.

### Decision Aid Assessment and Impact in Those Who Completed Surveys 1 and 2

#### Satisfaction With Decision Aid Design

Most parents (10/15; 67%) reported reading the DA “quite thoroughly” or “thoroughly from beginning to end,” with a median time of 25 minutes (range 15 to >60 minutes). All parents considered the length to be “about right,” 53% (8/15) reported that the DA was “very appealing” to look at, and 73% (11/15) mentioned that it was “very clearly” presented. In addition, 60% (9/15) parents were satisfied with the website format, while 33% (5/15) said they would also like a booklet, and 1 parent stated they would have liked a video.

#### Satisfaction With Content

The majority (13/15, 87%) of parents reported that the information in the DA was “balanced and fair,” and 13% (2/15) reported that the DA was in “favor of FP.” Most parents (12/15, 80%) felt that the information was “sufficiently detailed.” One parent found the DA to be confusing, while 87% (13/15) parents reported that it “clearly” or “very clearly” presented their child’s fertility choices. The majority (12/15, 80%) reported that the information would have been “quite” or “very” relevant when considering FP for their child.

#### Expectations of the Decision Aid

Overall, parents were “satisfied” (11/15, 73%) or “very satisfied” (4/15, 27%) with the DA. One parent, however, reported that the DA would not have helped them cope with their situation. The DA “met” (11/15, 73%) or “exceeded” (4/15, 27%) the expectations of all parents.

#### Emotional Impact of the Decision Aid

In this study, 47% (7/15) parents reported having “somewhat” thought about the information since reading the DA. In addition, 40% parents (6/15) reported feeling “a little” worried or concerned about the information. Themes emerging from open-ended responses related to concerns regarding future impacts of treatments on fertility with 1 parent commenting that she was “worried for my (child) as preservation was not an option for her” and another commenting that she was “not so much worried I guess, just sad,” indicating that worry was linked to concerns about the future impact of treatments on fertility.

#### Perceived Usefulness as a Decision-Making Tool

Overall, 86% (13/15) of parents reported that the DA would have been “helpful” or “very helpful” in helping them decide on their child’s treatment in general. In addition, 86% (13/15) reported that it would have been “helpful,” “very helpful,” or “extremely helpful” in making decisions about FP and would recommend the DA to other families facing an FP decision.

**Table 1:** Demographic characteristics of parents of children and adolescents with cancer.

Characteristics		Total parents (N=34)		Completed the pre-DA^a^ survey only (n=19)		Reviewed the DA and completed the pre- and post-DA surveys (n=15)		*P* value^b^	
Age in years, mean (SD); range		41.5 (11.1); 27-57		43.2 (8.1); 27-57		42.6 (7.0); 31-49		.28	
Age at child’s diagnosis in years, mean (SD); range		39.3 (11.2); 25-55		40.8 (7.7); 25-55		40.5 (6.7); 30-47		.19	
Decision regret score, mean (SD); range		15.6, (20.5); 0-95		16.8 (23.5); 0-95		16.5 (18.6); 0-50		.74	
**Country of birth, n (%)**									
	Australia		24 (71)		16 (67)		8 (33)		.84
	Other		10 (29)		3 (30)		7 (70)		
**Primary spoken language at home, n (%)**									
	English		29 (85)		18(62)		11(38)		.51
	Other		5 (15)		1 (20)		4 (80)		
**Relationship status, n (%)**									
	Married or de facto		25 (74)		12 (48)		13 (52)		.26
	Separated or divorced		7 (21)		5 (71)		2 (29)		
	Unknown		2 (6)		2 (100)		0 (0)		
**Highest level of education, n (%)**									
	≤Year 10		6 (18)		4 (67)		2 (33)		.04^c^
	Year 12		4 (12)		2 (50)		2 (50)		
	Technical and Further Education certificate or diploma		4 (12)		3 (75)		1 (25)		
	Bachelor’s degree		12 (35)		2 (17)		10 (83)		
	Postgraduate degree		7 (21)		7 (100)		0 (0)		
	Unknown		1 (3)		1 (100)		0 (0)		
**Employment status, n (%)**									
	Full time		9 (26)		2 (29)		7 (71)		.001^c^
	Part time		11 (32)		7 (64)		4 (36)		
	Self employed		2 (6)		1 (50)		1 (50)		
	Full-time or part-time student		1 (3)		0 (0)		1 (100)		
	Unemployed		7 (21)		6 (86)		1 (14)		
	Unknown		4 (12)		3 (75)		1 (25)		
**Occupation, n (%)**									
	Professional		15 (44)		6 (40)		9 (60)		.51
	Clerk or sales		3 (9)		2 (100)				
	Home duties		5 (15)		4 (80)		1 (20)		
	Other		11 (32)		7 (64)		4 (36)		
Parity, n (SD); range^d^		2.5 (1.1); 1-6		2.6 (1.4);1-6		2.3 (0.6);1-3		.32	
**Parents own past conception difficulties, n (%)**									
	Yes		5 (15)		1 (20)		4 (80)		.50
	No		29 (85)		18 (62)		11 (38)		
**Concerns regarding their child’s future fertility at diagnosis, n (%)**									
	Yes		23 (68)		12 (52)		11 (48)		.19
	No		8 (24)		5 (63)		3 (37)		
	Unsure		3 (9)		2 (67)		1 (33)		
**Recalled a fertility discussion, n (%)**									
	Yes		29 (85)		16 (55)		13 (45)		.85
	No		5 (15)		3 (60)		2 (40)		
**Clinician involved in fertility discussion, n (%)**									
	Oncologist		12 (35)		7 (58)		5 (42)		.10
	Gynecologist		7 (21)		4 (57)		3 (43)		
	Oncologist + gynecologist or endocrinologist or nurse		7 (21)		4 (57)		3 (43)		
	Endocrinologist		0 (0)		0 (0)		0 (0)		
	Nurse		2 (6)		1 (50)		1 (50)		
	Social worker		2 (6)		1 (50)		1 (50)		
	Unknown		4 (12)		2 (50)		2 (50)		

^a^DA: decision aid.

^b^*t* test (two-tailed) between parents who completed only the pre-DA survey only and those who completed both pre- and post-DA surveys.

^c^Significant at *P* ≤.05.

^d^The number of children the parents of patients have had.

**Table 2:** Characteristics of childhood and adolescent patients with cancer.

Characteristics		Total children and adolescents (N=34)		Those whose parents completed the pre-DA^a^ survey only (n=19)		Those whose parents reviewed the DA and completed the pre-and post-DA surveys (n=15)		*P* value^b^
Age in years, current mean (SD); range		9.9 (6.2); 1.5-19.6		9.9 (6.2); 1.8-19.6		9.3 (6.4); 1.5-19.2		.88
Age at diagnosis in years, mean (SD); range		7.7 (5.9); 1.0-17.2		7.7 (6.0); 1.0-17.2		7.4 (5.9); 1.0-17.2		.58
**Pubertal status at diagnosis, n (%)**								
	Prepubertal		22 (65)		12 (55)		10 (45)	.63
	Postpubertal		12 (35)		7 (58)		5 (42)	
**Diagnosis, n (%)**								
	Leukemia		10 (29)		4 (40)		6 (60)	.38
	Rhabdomyosarcoma		5 (15)		2 (40)		3 (60)	
	Ewing’s Sarcoma		5 (15)		4 (80)		1 (20)	
	Central nervous system		3 (9)		2 (67)		1 (33)	
	Hodgkin’s Disease		2 (6)		1 (50)		1 (50)	
	Osteosarcoma		3 (9)		2 (67)		1 (33)	
	Other solid cancers		5 (15)		3 (60)		2 (40)	
	Non-Hodgkin’s		1 (3)		1 (100)		0 (0)	
**Estimated risk of infertility, n (%)**								
	Low		5 (15)		1 (20)		4 (80)	.04
	Medium		14 (41)		8 (57)		6 (43)	
	High		15 (44)		10 (67)		5(33)	
**Type of fertility preservation procedures, n (%)**								
	Ovarian tissue cryopreservation		8 (24)		6 (75)		2 (25)	.68
	Ovarian tissue cryopreservation + gonadotropin-releasing hormone agonist + oocytes		2 (6)		1 (50)		1 (50)	
	Testicular tissue cryopreservation		10 (29)		5 (50)		5 (50)	
	Semen cryopreservation		3 (9)		2 (67)		1 (33)	
	No procedure		11 (32)		5 (45)		6 (55)	

^a^DA: decision aid.

^b^*t* test (two-tailed); significant at *P≤*.05.

Of the 8 parents who completed the VCE, half reported that it would have been “satisfactory” in helping them decide, while the other half reported it would have been “very helpful.” Reasons cited for not completing the VCE included time constraints, that the parent believed it was irrelevant to their situation, or that they had technical issues with the use of the website.

### Improvements in Knowledge and Understanding

In this study, 74% (11/15) parents reported that only some of the information was new to them. The remaining parents reported that either “most” (2/15; 13%) or “none” (2/15; 13%) of the information was new to them. Overall, parents reported the DA helped improve their understanding of cancer treatments, infertility, and FP procedures to some degree (Multimedia Appendix 3).

In addition, 14 parents answered the FP knowledge scale pre- and post-DA review. Prior to the review, parents answered an average of 5.21 (SD 1.66; range 1-8) out of 10 FP knowledge questions correctly. Knowledge scores improved by 1.50 to an average of 6.71 correctly answered questions after reviewing the DA (Table 3); this was a significant (*P*<.04) increase in the number of correct responses overall. Prior to reviewing the DA, 21% (3/14) parents scored >70% on the FP knowledge scale; this increased to 64% (9/14) after DA review.

### Expectations of the Fertility Preservation

**Figure 4:**
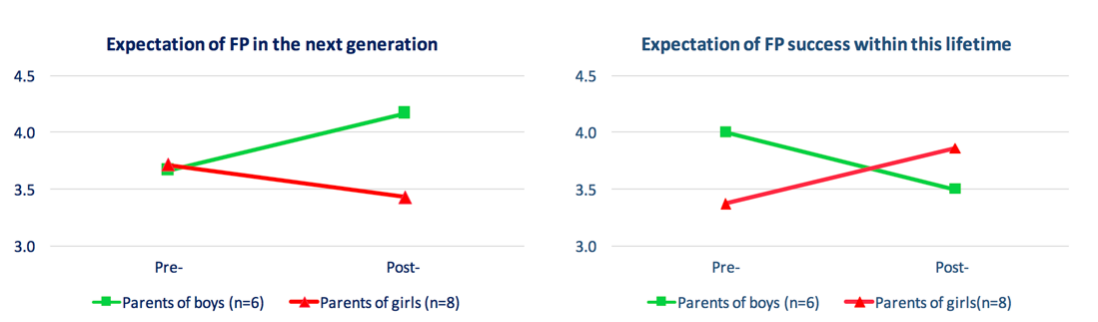
Parents' expectations of fertility preservation success within this lifetime and the lifetime of the next generation. FP: fertility preservation; 5: Strongly agree; 4: Agree; 3: Neither agree nor disagree; 2: Disagree; 1: Strongly disagree.

The expectation of the FP success was asked in general, not relating specifically to their child and encompassed all FP procedures, not just experimental procedures. Notably, 11 parents reported their expectations of the future success of FP procedures. The majority “agreed” or “strongly agreed” (8/15; 73%) that FP would be successful in their lifetime. Similarly, 73% (11/15) responded that they “agreed” or “strongly agreed” that FP will be successful within the lifetime of their child. This decreased to 46% (7/15) after DA review. The change in expectations varied between parents of boys and girls (Figure 4). Expectations of success in this lifetime decreased in parents of boys and increased in parents of girls. Conversely, expectations of success in the next generation increased for parents of boys and decreased in girls.

### Decision Regret

In this study, 14 parents completed the Decision Regret Scale (Table 3). The mean regret score pre-DA review was 16.5 (SD 18.6; range 0-50), and post-DA review was 18.5 (SD 19.4; range 0-50). There was a nonsignificant increase in scores across all parents of 1.9 points (4.2-point increase in parents of boys and 0 in parents of girls; *P*=.52).

### Clinician Review of the Decision Aid

#### Usability and Content Usefulness and Satisfaction of the Decision Aid Design

All clinicians reported that they would recommend the DA to patients. When asked for their thoughts on the DA, three main themes emerged from the comments: (1) the DA was well designed and easy to use; (2) the DA was a good information source; and (3) there is a need for more information and resources for patients and parents beyond the DA.

#### Design, Usability, and Content

Clinicians reported satisfaction with the design and usability of the DA website, commenting that it was an “excellent and well-structured” resource. In addition, the DA was regarded as a valid and relevant source of information for clinicians, patients, and their families with one clinician commenting that “I found it useful as a resource prior to meeting with a patient.”

#### Perceived Need for Information and Patient Resources

In this study, 36% (4/11) of the interviewed clinicians highlighted a lack of patient and parent resources regarding infertility, FP procedures, and processes. One clinician commented that she had “observed more and more adolescents, especially boys” making FP decisions, noting that there are very few resources tailored toward adolescents and parents of adolescents.

**Table 3:** Change in the parental fertility preservation-related knowledge and decision regret pre- and postdecision aid (n=15).

Change		Pre-DA^a^ mean score (SD); range		Post-DA mean score (SD); range		Mean change score		SE		95% CI		*t* ^b^		Degree of freedom		*P* value	
**Fertility preservation knowledge**																			
	All parents (n=14)		5.21 (1.66); 1-8		6.71 (1.94); 3-10		1.50		0.66		0.07 to 2.93		2.27		13		.04^c^
	Parents of boys (n=6)		5.17 (1.46); 4-8		6.33 (2.13); 3-10		1.17		1.20		0.19 to 1.90		0.98		5		.37
	Parents of girls (n=8)		5.25 (1.79); 1-7		7 (1.73); 4-10		1.75		0.80		0.13 to 3.63		2.20		7		.06
**Decision regret scale scores**																			
	All parents (n=14)		16.5 (18.6); 0-50		18.5 (19.4); 0-50		1.9		5.17		–9.51 to 4.67		0.64		13		.54
	Parents of boys (n=6)		5.8 (12.0); 0-30		10.0 (16.7); 0-40		4.2		3.75		–13.79 to 5.46		1.11		5		.32
	Parents of girls (n=8)		25.7 (19.0); 0-50		25.7 (19.7); 0-50		0		4.76		–11.64 to 11.64		0.00		7		1.0

^a^DA: decision aid.

^b^*t* test (two-tailed).

^c^Significant at *P≤*.05.

## Discussion

In this pilot study, we evaluated the parental and clinical acceptance of the first FP DA for parents of children and adolescents with cancer. Our data suggest that the DA was acceptable, did not increase parental concern, and would be useful for parents making an FP decision. Furthermore, initial testing suggests the DA increases FP-related knowledge. Parents and clinicians would recommend this tool to others faced with a similar decision. However, we acknowledge the high dropout in this study. These results support further formal evaluation of the DA in a larger prospective trial, prior to the implementation of the DA as a decision-support tool in clinical practice.

The DA was positively received by parents who viewed it. Parents felt that the DA provided unbiased information in an easy-to-read format and was relevant to their situation. Of note, 13% (2/15) parents felt that the DA favored FP. The theoretical framework behind DA development is that it should not favor a particular option but provide a balanced view so that users can make fair decisions [21]. Most of the cohort had undergone an FP procedure, and our DA presented a large amount of information concerning FP procedure outcomes, which may have contributed to the impression of some parents that it favored FP.

One of the primary purposes of our DA was to increase parents’ fertility-related knowledge scores in parents despite parents already having experience with FP. These results should be interpreted cautiously because of small numbers. However, in parents with no or little FP awareness, we hope there would be a more significant impact on knowledge, as has been demonstrated in other health-related DA studies [21,29].

A key component of informed decision making is an understanding of the likelihood of the possible outcomes and the associated risks and benefits [13]. Interestingly, post-DA review parents’ expectations of FP had changed. Expectations of the success of experimental procedures “in this lifetime” decreased for parents of boys and increased for parents of girls; this change may reflect a better understanding of the technologies as they currently stand, considering that ovarian tissue cryopreservation is being used to achieve pregnancy, but testicular tissue cryopreservation has not yet been successfully used in humans. That parents had perhaps more realistic expectations of FP is important in that (1) they may have a more accurate perception of the risks and likely future successes of FP; (2) that improved perception may lead to better-quality decision making, thereby decreasing the risk of future decision regret; and (3) there may be scope for this DA to improve informed consent in clinical practice.

The DA had an emotional impact on some parents, with 40% (6/15) reporting feeling “a little” worried or concerned after reviewing the DA; this was somewhat expected as the DA was a comprehensive fertility resource and the additional information may have raised issues that were not previously discussed with parents at the time of decision making or may have since been forgotten. One participant stated that the information in the DA gave them a more realistic understanding of their child’s fertility risk; this highlights the importance of ensuring that parents are well informed at the time of diagnosis and receive adequate fertility counseling during their posttreatment care [30].

The DA did not increase decision regret in parents; this may reflect that the DA confirmed that parents made the right decision. Ultimately, it does not appear that the DA increases distress, and thus, it is likely to be suitable for use in parents of newly diagnosed children adolescents. Notably, decision regret was measured shortly after DA review. While it was not evident at that time, it is possible that regret may increase months or years later [31], depending on the state of reproductive technologies when the patient wishes to conceive; this has yet to be explored and is an area for further research.

A novel feature of this DA is the rapid feedback provided through the Web-based VCE, which provided a visual representation of the importance of FP to participants, based on the responses provided to a series of questions. To the best of our knowledge, this is the first report of a VCE that functions in this way. All parents who completed the VCE reported that it would have been helpful to some extent when making the decision. Although our data did not provide the information to ascertain whether this was a result of the ease of the click-through VCE, the visual result, or both, it does suggest that a Web-based tool may have merit. Although these data are positive, only 53% (8/15) completed the VCE. Reasons for noncompletion included time constraints, inaccessibility, and unclear instructions. A post-hoc consumer review revealed that the link name for the VCE was confusing. Other studies have reported varying rates of the VCE completion in pilot studies and suggest that what is reported in pilot studies is not reflective of what happens in prospective studies [32]; thus, prospective evaluation is needed.

Although our findings support the utility of a fertility DA for parents of children and adolescents with cancer, our study had inherent limitations. As with most small samples, care must be taken with interpretation of results, as they may not be generalizable. Parents were asked to reflect on their FP decision-making process, which could have been up to 5 years previously. It was not possible to capture the lived experience, and parents may have been biased by intervening events. In addition, study measures were limited by a retrospective sample. Therefore, it was not possible to measure the decisional conflict, a measure of uncertainty and a key factor affecting decision making [33]. However, this study design is an appropriate and necessary way to assess DA acceptability and usability prior to prospective evaluation.

Dropout in this study was higher than expected, resulting in a small sample size. While parents indicated they were keen to participate, many noted that the high time demands of the study and had limited time to engage in a detailed review of the DA. It is likely that in a study of parents making prospective decisions in real time, a higher proportion of parents would wish to actively review the DA. Previous pilot DA studies have shown similar sample sizes to be sufficient to test acceptability and usability of the tools prior to prospective evaluation [32,34].

Overall, clinicians reported satisfaction with the DA design and most importantly would recommend the DA to parents facing a fertility-preservation decision. The resource was regarded as valid and relevant, and interestingly was “a useful resource prior to meeting with a patient.” Research has shown that even with the best intentions, clinicians struggle to convey information and potential risks to patients in language that is easily comprehensible [14]. In the future, this plain language resource could be used to support clinician education and potentially improve fertility-preservation counseling. Lastly, clinicians reported a lack of resources to support children and adolescents in the shared decision-making process. Perhaps the development of an adolescent and young adult FP DA or toolkit may address this gap.

There is a growing body of evidence highlighting the importance of information provision regarding the risks to fertility from cancer treatments and potential FP options. Information provision is important at the time of diagnosis prior to treatment and the potential harm to reproductive tissues [8]. Information regarding cancer treatments, fertility, and potential FP procedures is complex and may be difficult to comprehend [35], especially given the stress of a new cancer diagnosis and the short timeframes in which patients and their parents are required to make decisions. This DA is acceptable and relevant to parents and may assist families who are actively engaged in making an FP decision. Results warrant evaluation in prospective studies, which can assess outcome measures such as decisional conflict as well as decision regret.

As the health care user and provision landscape are rapidly changing, it is increasingly important for health care tools to evolve to further improve patients’ interactions with health care systems, clinical teams, and improve participation and satisfaction with shared decisions. This novel study has developed and preliminarily assessed the first, Web-based FP DA for parents of children and adolescents with cancer. This research has shown the DA to be acceptable to parents who have previously made an FP decision for their children and adolescents with cancer without causing distress. This study adds to the growing pool of research regarding pediatric FP, DA evaluation, and parental (proxy) decision making.

## Appendix

Multimedia Appendix 1 Fertility decision aid content.

Multimedia Appendix 2 Pre- and postdecision aid survey measures.

Multimedia Appendix 3 Improved understanding of fertility preservation in parents’ postdecision aid review; n=15 (%).

## Abbreviations

DA: decision aid
FP: fertility preservation
VCE: Values Clarification Exercise
