# Supplementary material for: Fertility Preservation in Children and Adolescents With Cancer: Pilot of a Decision Aid for Parents of Children and Adolescents With Cancer
Source: JMIR Pediatr Parent. 2018 Nov 28;1(2):e10463. doi: 10.2196/10463 (PMC6715396; doi:10.2196/10463)
Supplement: Multimedia Appendix 1 [file pediatrics_v1i2e10463_app1.pdf]

Multimedia Appendix 1. Fertility decision aid content.

Page /Chapter	Contents
"About Us"	Gives a brief overview of who created the website, what the website is about, who the information in the decision aid is designed for.
"Some things to think about"	Discusses why fertility is important to some cancer survivors. Also includes some information about there being no right or wrong decision. A brief section is included on talking to children and adolescents about fertility.
"Fertility preservation"	Overview of fertility preservation
"Fertility and age"<br>/Fertility and age<br>/Fertility as we grow	Describes fertility over the lifetime; puberty and the onset of fertility; natural fertility decline. (Divided into male and female pages)
"Fertility and cancer"<br>/Fertility and cancer<br>/Cancer treatments	Describes how different cancers and cancer treatments can affect fertility. Includes information and statistics on fertility post-cancer treatment, explains different risks, and includes more specific information about chemotherapy, radiotherapy and surgery. (Divided into male and female pages)
"Fertility preservation"<br>/Fertility preservation procedures<br>/Freezing ovarian tissue<br>/Oocyte preservation<br>/Zoladex injections<br>/Freezing testicular tissue<br>/Freezing sperm<br>/Other options	Section gives an overview of the available fertility preservation procedures. Descriptions include explanation of potential risks and benefits, the procedure, how reproductive technologies are used, and the costs (current and future). Diagrams and infographics are included to show probabilities of outcomes and future pregnancies or live births from the procedure. Links to consumer videos are included also. (Divided into male and female pages)
"Talking to your child"	Overview of talking to children and adolescents about fertility and fertility preservation. An explanation of why doctors/health-care team may involve children and adolescents in the discussion.
"Values clarification"	Interactive values clarification tool. Allows patients to weigh the importance of statements

	“for” or “against” fertility preservation. Statements are divided by pubertal status and age.
“Other useful information”	Links to other resources about fertility, cancer, and fertility preservation that are available on the internet or in clinics. These resources are already used in clinical practice.
“Glossary”	Definitions of medical concepts and words in “lay” terms.
“Contact us”	Contact links for the research team, contact details of the lead investigator. Acknowledgments.
