# Supplementary material for: Fertility Preservation in Children and Adolescents With Cancer: Pilot of a Decision Aid for Parents of Children and Adolescents With Cancer
Source: JMIR Pediatr Parent. 2018 Nov 28;1(2):e10463. doi: 10.2196/10463 (PMC6715396; doi:10.2196/10463)
Supplement: Multimedia Appendix 2 [file pediatrics_v1i2e10463_app2.pdf]

Multimedia Appendix 2. Pre- and post-decision aid survey measures.

Measure	Pre-DA[a]	Post-DA
**Demographic information:** Age at diagnosis, current age, relationship status, parity, education, employment status, and reproductive and social history.	□	
**Clinician Discussion, Referral and Procedure:** Six open-ended items recorded fertility discussion recall, referrals, decision-making roles, and perception of child's understanding of infertility risk and fertility preservation procedures.	□	
**Knowledge Scale:** A 10-item Fertility Preservation knowledge scale was purposefully designed, adapted from a scale previously used [23]. Responses included: "yes" (1), "no" (0) and "not sure" (0). The scores for each item were summed to give a final score out of 10. Higher scores indicated greater knowledge with reasonable internal validity (α = 0.54).	□	□
**Decision Regret Scale:** The validated 5-item Decision Regret Scale [24] was included to measure changes in distress or remorse about the health-care decision (in this instance FP[b]) after using the DA. Responses to the scale were in relation to DR regarding previous fertility treatment and care. For this study, DR scores <30 were classified as low, while scores of 30 or above were classified as moderate/high [33]. In addition, 2 free text items allowed parents to give explanations as to why or why not they regretted their decision.	□	□
**Expectation of FP Success:** Two items assessed parental belief that FP would be successful (a) in their lifetime, or (b) in their child's lifetime. Response options included "strongly disagree," "disagree," "neither agree nor disagree," "agree," and "strongly agree."	□	□
**Perceived FP knowledge and understanding:** Seven Likert-type questions assessed to what extent parents thought the DA would have improved their knowledge about fertility related issues when their child was first diagnosed. Responses included: "a lot" (5), "quite a bit" (4), "somewhat" (3), "a little" (2), and "not at all" (1).		□
**Satisfaction with the design of the DA:** Five Likert-type questions assessed satisfaction with the design of the DA. The questions addressed the extent to which the DA was read ("thoroughly from beginning to end," "quite thoroughly," "just parts that I felt were relevant to me," "briefly"), time taken ("<15 mins," "20 mins," "30 mins," "40 mins," "50 mins" ">1 hour"), perceptions about length ("much too short," "too short," "about right," "too long," and "much too long"), appeal ("very," "somewhat," "not very," "not at all"), clarity of presentation ("very," "somewhat," "not very," "not at all"). An additional free-text item asked if they were happy with the website format.		□

**Satisfaction with DA content:** Six questions assessed satisfaction with content in terms of balance (“for fertility preservation,” “completely balanced,” “against fertility preservation”), amount of information (“Far too little,” “too little,” “about right,” “too much,” “far too much”), need for greater explanation (“yes” or “no”), confusing (“yes” or “no”), how clearly the child’s fertility choices were explained (“very clearly,” “clearly,” “unclearly,” “very unclearly”), and relevance of information at the time of decision-making (“very,” “quite,” “not very,” “not at all”).		□
**Expectations of the DA:** Four Likert-type questions assessed clarity around explanation of FP choices (“very clearly,” “clearly,” “unclearly,” “very unclearly”), the degree to which the DA helped with coping (“very much so,” “quite a bit,” “somewhat,” “a little,” “not at all”), satisfaction with the information (“very satisfied,” “satisfied,” “dissatisfied,” “very dissatisfied”), and if the DA met expectations (“greatly exceeded,” “exceeded,” “met,” “I was disappointed,” “not at all”).		□
**Emotional impact of the DA:** Two items assessed how much the parent had thought about the content in the DA, and whether reading the DA made them feel worried or concerned. Responses included: “not at all,” “a little,” “somewhat,” “quite a bit,” and “very much so.”		□
**Perceived use as a decision-making tool:** Five 4-point Likert-type items, adapted from a DA development study, assessed perceived usefulness of the DA as decision-making too. Specifically, relevance (“not at all,” “not very,” “quite,” and “very”), helpful with coping (“not at all,” “a little,” “somewhat,” “quite a bit,” and “a lot”), clarity of information (“very clearly,” “clearly,” “unclearly,” or “very unclearly”), helpful in making the decision and helpfulness in reaching a decision (“extremely unhelpful,” “very unhelpful,” “helpful,” “very helpful,” “extremely helpful”). One item assessed if parents had used the VCE, with a free text option to provide reasons why they had not. One item assessed if completing the exercise would have been helpful to decide (“extremely unhelpful,” “very unhelpful,” “unhelpful,” “satisfactory,” “very satisfactory,” “extremely satisfactory”). Two Likert-type items assessed the extent to which the DA would have been helpful in deciding about their child’s fertility management and about a fertility preservation procedure (“extremely unhelpful,” “very unhelpful,” “unhelpful,” “helpful,” “very helpful,” “extremely helpful”). One item assessed whether they would recommend the DA to others (“yes,” “not sure,” “no”).		□

[a]DA: decision aid.
[b]FP: fertility preservation.
