# Supplementary material for: Fertility Preservation in Children and Adolescents With Cancer: Pilot of a Decision Aid for Parents of Children and Adolescents With Cancer
Source: JMIR Pediatr Parent. 2018 Nov 28;1(2):e10463. doi: 10.2196/10463 (PMC6715396; doi:10.2196/10463)
Supplement: Multimedia Appendix 3 [file pediatrics_v1i2e10463_app3.pdf]

**Multimedia Appendix 3.** Improved understanding of fertility preservation in parents' post-decision aid review; n=15 (%).

| **To what extent do you think the Decision Aid would have improved your understanding of the…** | **A lot n (%)** | **Quite a bit n (%)** | **Somewhat n (%)** | **A little n (%)** | **Not at all n (%)** |
|---|---|---|---|---|---|
| *…impact of different cancer treatments on fertility* | 0 (0) | 4 (27) | 6 (40) | 5 (33) | 0 (0) |
| *…different fertility options available to children and adolescents* | 0 (0) | 6 (40) | 5 (33) | 4 (27) | 0 (0) |
| *…pros of different fertility options available to children and adolescents* | 0 (0) | 3 (20) | 7 (47) | 5 (33) | 0 (0) |
| *…cons of different fertility options available to children and adolescents* | 0 (0) | 5 (33) | 6 (40) | 3 (20) | 1 (7) |
| *…impact of different fertility treatments on your child and family* | 0 (0) | 2 (13) | 9 (60) | 1 (7) | 3 (20) |
| *…impact of different fertility treatments on your child's other medical treatment* | 0 (0) | 3 (20) | 7 (47) | 5 (33) | 0 (0) |
| *…impact of different fertility treatments on the future outcome of the disease your child had* | 0 (0) | 3 (20) | 6 (40) | 2 (13) | 4 (27) |
